# In silico-designed lignin peroxidase from *Phanerochaete chrysosporium* shows enhanced acid stability for depolymerization of lignin

**DOI:** 10.1186/s13068-018-1324-4

**Published:** 2018-12-10

**Authors:** Le Thanh Mai Pham, Hogyun Seo, Kyung-Jin Kim, Yong Hwan Kim

**Affiliations:** 1 School of Energy and Chemical Engineering, UNIST, 50 UNIST-gil, Ulju-gun, Ulsan, 44919 Republic of Korea; 2 School of Life Sciences (KNU Creative BioResearch Group), KNU Institute for Microorganisms, Kyungpook National University, Daehak-ro 80, Buk-gu, Daegu, 41566 Republic of Korea

**Keywords:** *Phanerochaete chrysosporium*, Lignin peroxidase, Lignin degradation, Salt bridges, Acid stability, In silico design

## Abstract

**Background:**

The lignin peroxidase isozyme H8 from the white-rot fungus *Phanerochaete chrysosporium* (LiPH8) demonstrates a high redox potential and can efficiently catalyze the oxidation of veratryl alcohol, as well as the degradation of recalcitrant lignin. However, native LiPH8 is unstable under acidic pH conditions. This characteristic is a barrier to lignin depolymerization, as repolymerization of phenolic products occurs simultaneously at neutral pH. Because repolymerization of phenolics is repressed at acidic pH, a highly acid-stable LiPH8 could accelerate the selective depolymerization of recalcitrant lignin.

**Results:**

The engineered LiPH8 was in silico designed through the structural superimposition of surface-active site-harboring LiPH8 from *Phanerochaete chrysosporium* and acid-stable manganese peroxidase isozyme 6 (MnP6) from *Ceriporiopsis subvermispora.* Effective salt bridges were probed by molecular dynamics simulation and changes to Gibbs free energy following mutagenesis were predicted, suggesting promising variants with higher stability under extremely acidic conditions. The rationally designed variant, A55R/N156E-H239E, demonstrated a 12.5-fold increased half-life under extremely acidic conditions, 9.9-fold increased catalytic efficiency toward veratryl alcohol, and a 7.8-fold enhanced lignin model dimer conversion efficiency compared to those of native LiPH8. Furthermore, the two constructed salt bridges in the variant A55R/N156E-H239E were experimentally confirmed to be identical to the intentionally designed LiPH8 variant using X-ray crystallography (PDB ID: 6A6Q).

**Conclusion:**

Introduction of strong ionic salt bridges based on computational design resulted in a LiPH8 variant with markedly improved stability, as well as higher activity under acidic pH conditions. Thus, LiPH8, showing high acid stability, will be a crucial player in biomass valorization using selective depolymerization of lignin.

**Electronic supplementary material:**

The online version of this article (10.1186/s13068-018-1324-4) contains supplementary material, which is available to authorized users.

## Background

Depolymerization and utilization of lignin are essential steps in carbon recycling in terrestrial ecosystems. Conversion of lignin into value-added chemicals is a hot topic in the biorefinery field, which drives further development of lignin degradation processes using chemical, biological and biochemical catalysts [1].

An efficient, natural process for the accelerated degradation of lignin has been developed by white-rot fungi that belong to Basidiomycetes [2]. To efficiently degrade lignin, white-rot fungi evolved unique ligninolytic peroxidases, such as manganese peroxidase (MnP), lignin peroxidase (LiP) or the versatile peroxidase (VP), showing unique characteristics, such as mediator utilization and surface-active sites to increase redox potential. LiPs and VPs can directly oxidize nonphenolic lignin compounds through surface-active sites [3, 4]. Notably, lignin peroxidase isozyme H8 (LiPH8) from the white-rot fungus *Phanerochaete chrysosporium* directly interacts with lignin macromolecules, a finding which was supported by kinetic analysis of its binding affinity [5]. However, quantitative detection of phenolic products or a significant decrease in lignin molecular weight has not been reported for in vitro depolymerization of lignin by LiPH8. It is thought that repolymerization of degraded lignin fragments may spontaneously occur, which could pose a barrier to in vitro depolymerization. In the oxidative depolymerization of lignin, one of the challenges is to control the reactivity of oxygen-based radical species, thereby limiting the problem of recombination/repolymerization of lignin fragments. The pH of the reaction is one of the routes for addressing this problem [6, 7]. In the culturing of *P. chrysosporium*, the production of organic acids resulted in a pH below or equal to pH 2, which is critical for in vivo degradation of lignin [8]. Therefore, the poor acid stability of native LiPH8 is believed to hamper effective in vitro depolymerization of lignin. Active and acid-stable LiPH8 is, thus, urgently required. Work to engineer other ligninases, such as MnPs and VPs, for acidic stability has been reported [7]. However, there are no reported studies of LiPH8, even though LiPH8 has the strongest oxidation power for the depolymerization of lignin.

The conformational stability of a protein is vital to its function and can be affected by noncovalent interactions, such as hydrogen bonds and salt bridges [9–11]. Although disulfide bonds contribute increased structural stability to folded proteins at optimal temperatures compared to that contributed by noncovalent interactions, however introducing artificial disulfide bridges has occasionally resulted in protein aggregation owing to oxidation-induced intermolecular disulfide bonds [12]. In some cases, salt bridges can be key interactions for sustaining the structure of a protein, such as disulfide bonds [13]. The impact of a salt bridge on the structure of a protein strongly depends on its relative location, orientation and distance between interacting residues, which makes designing a salt bridge network to increase protein stability challenging.

Evolution of MnPs into LiPs parallels the removal of Mn^2+^ binding sites and the creation of surface tryptophan residues, which accelerates interaction with the bulky structure and oxidation of high-redox-potential substrates, such as lignin [14]. Note that this evolution might unexpectedly result in poor acid stability of the modern LiP. It was also found that various white-rot fungi, such as *P. chrysosporium* [15, 16], *Trametes* sp. [17–19], *Coriolopsis byrsina, Phellinus rimosus* and *Lentinus* sp. [19] possess LiP isozymes which are not stable under extremely acidic conditions (e.g., pH values lower than pH 3.0). Even though LiPs and MnPs share a similar overall structure, as both belong to members of the peroxidase family, MnPs found in fungi, such as *Ceriporiopsis subvermispora* and *Pleurotus ostreatus*, exhibit relatively higher stability under acidic pH conditions [7, 20]. MnP6 from *C. subvermispora* is exceptionally resilient, as it can retain its activity under extremely acidic conditions, such as pH 2.0 [4]. Four of the five disulfide bridges in MnP6 are conserved in the LiPH8 structure. There is an extra disulfide bridge that can stabilize the extraordinarily long *C*-terminus of MnP6 (i.e., when compared with other ligninases). We concluded that the considerable acid stability observed could be a result of several noncovalent interactions, such as salt bridges and hydrogen bonding networks. Moreover, these kinds of interaction could help to maintain protein conformation even at high concentrations of protons [20].

In this study, we proposed an in silico-based strategy to design active LiPH8 variants for increased stability in intensively acidic environments. Introduction of new strong salt bridges at effective locations and optimized interactions between charged residues and their environments were vital for active and stable LiP at acidic pH. Probing for existing noncovalent interactions, especially salt bridges, using a molecular dynamics (MD) simulation of the solvated structure under the desired conditions and calculating the Gibbs free energy of the variant were valuable tools for creating an acid-stable LiP variant. Protein X-ray crystallography was also employed to verify the existence of the designed salt bridges introduced between interacting residues of the LiPH8 variants.

## Materials and methods

### Materials

Hydrogen peroxide, hemin, oxidized glutathione, ampicillin, isopropyl-β-d-thiogalactopyranoside, 2,2′-azino-bis(3-ethylbenzothiazoline-6-sulfonic acid) diammonium salt (ABTS), guanidine hydrochloride, dibasic potassium phosphate, citric acid, trizma^®^ hydrochloride and veratryl alcohol (VA) were purchased from the Sigma Chemical Co., South Korea and were used without any further purification. Veratrylglycerol β-guaiacyl ether (VE dimer) ether as a model dimeric lignin at 97% purity was purchased from AstaTech, Inc., USA.

### Hardware and software specifications

All the molecular modeling studies were conducted on a workstation running the Windows 10 operating system and equipped with an Intel Xeon E5-2620 v3 CPU, 32 GB of RAM, and a high-end NVIDIA graphics card. For MD simulations, MD trajectory analysis and structural analysis were conducted using Discovery Studio Client v18.1.0.17334 (Dassault Systems Biovia Corp.)

### Protein expression and purification

The synthetic LiPH8 gene, including the seven-residue pro-sequence, was synthesized by the Bioneer Company (South Korea). The gene-coded protein sequence, which was retrieved from a previously published report [21] (UniProtKB entry: P06181), was cloned into the commercially available ampicillin-resistant *E. coli* expression vector pET21b(+) (Novogene, USA) via the *Nde*I and *Eco*RI restriction sites (denoted as pET-LiPH8). The native gene pET-LiPH8 was expressed in *E. coli* strain BL21 (DH3).

The mutations were introduced into the LiPH8 gene by PCR using the expression plasmid pET-LiPH8 as a template and primers containing the desired mutations, designed as previously reported [22]. Detailed information of the synthesized oligonucleotide primers containing the desired mutations, with each primer complementary to the opposite strand of the vector, is reported in Additional file 1: Table S1. The PCR (50-μL reaction volume) was carried out in a Bio-Rad (California, USA) MyCycler using 50 ng of template DNA, 0.5 μM forward and reverse primers, and 2.5 units of *Pfu* DNA polymerase (BioNeer, South Korea) in 1× FailSafe PreMix G (Lucigen, USA). Reaction conditions included (i) a start cycle of 5 min at 95 °C; (ii) 15 cycles of 1 min at 95 °C, 50 s at 60 °C, and 15 min at 68 °C; and (iii) a final cycle of 15 min at 68 °C. The wild-type and mutated genes were expressed as inclusion bodies, reactivated through refolding and purified as previously reported [21]. After purification, enzymes were stored in acetate buffer 10 mM, pH 6.0. The UV–visible spectrum of native LiPH8 and its variants were recorded in the range of 250–600 nm to check the correct incorporation of heme into the protein. Enzyme concentration was determined from the absorbance of the Soret band (*Ɛ*_409_ = 168 mM^−1^ cm^−1^) [21].

### Crystallization, data collection, and structure determination

The purified protein was initially crystallized by the hanging-drop vapor-diffusion method at 20 °C using commercially available sparse-matrix screens from Hampton Research and Emerald BioSystems. Each experiment consisted of mixing 1.0 μL of the protein solution (8 mg/mL in 10 mM succinate buffer at pH 6.0) with 1.0 μL of the reservoir solution and then equilibrating the mixture against 0.5 mL of the reservoir solution. LiPH8-variant crystals were observed under several crystallization screening conditions. After several optimization steps using the hanging-drop vapor-diffusion method, the best-quality crystals appeared after 7 days using a reservoir solution consisting of 16% PEG 6000, which reached maximal dimensions of approximately 0.3 × 0.1 × 0.1 mm. For cryo-protection of the crystals, a solution of 30% glycerol suspended in the reservoir solution was used. Data were collected on a beamline 7A using a Quantum 270 CCD detector (San Diego, CA, USA) at a wavelength of 0.97934 Å. The LiPH8 variant crystal diffracted to a resolution of 1.67 Å. The data were then indexed, integrated, and scaled using the HKL2000 program [23]. Crystals of the LiPH8 variant belonged to the space group P21 with unit cell dimensions of *a*: 41.2 Å; *b*: 99.6 Å; *c*: 48.3 Å; *α*, *γ*: 90.0; and *β*: 113.9. With one LiPH8 variant molecule per asymmetric unit, the crystal volume per unit of protein mass was approximately 2.46 Å^3^ Da^−1^, which corresponded to a solvent content of approximately 50.11% [24]. The structure of the LiPH8 variant was solved by the molecular replacement method using MOLREP [25] with the original LiPH8 structure (PDB code 1B80) as a search model. Model building was performed using the WinCoot program [26] and refinement was performed with REFMAC5 [27]. The refined models of the LiPH8 variant were deposited in the Protein Data Bank (PDB CODE 6A6Q).

### MD simulations

The crystallized structures of MnP6 from *C. subvermispora* (PDB 4CZN), native LiPH8 from *P. chrysosporium* (PDB 1B80) and mutated LiPH8 were applied with a CHARMM force-field to assign atom types. The calculations of the protein ionization and residue pKa values in this study were based on the fast and accurate computational approach to pH-dependent electrostatic effects in protein molecules [28]. The titratable states of the amino acids were assigned based on a calculation of protein ionization and residue pK_a_ protocol at pH 2.5. The structures were solvated by adding water molecules (6834, 8393, and 7743 water molecules for MnP6, native LiPH8 and LiPH8 variant, respectively) and counterions (NaCl 0.1 M) with periodic boundary conditions. The solvated structures were subjected to energy minimization with a Smart Minimizer including 1000 steps of Steepest Descent with an RMS gradient tolerance of 3, followed by Conjugate Gradient minimization. Then, “The Standard Dynamics Cascade” protocol was applied as a set of simulation procedures to the minimized structures. This protocol performed a set of heating (10 ps), equilibration (1 ns) and production (2 ns) using CHARMM force-field with SHAKE constraint. Snapshots were collected during the last 2 ns of the MD simulation (2-ps interval). Then, the “Analyze Trajectory” protocol was applied and involved root-mean-square deviations (RMSD) of backbone atoms relative to the corresponding crystal structures as a function of time, and the per-residue root-mean-square fluctuation (RMSF) was performed via the Discovery Studio package. Potential ionic bonds (salt bridges) were detected when a positively charged nitrogen atom of lysine (NZ) or arginine (NH1, NH2) or positively charged histidine (HIP: ND1 NE2, both protonated) was found to be within 4.0 Å of a negatively charged oxygen atom of glutamate (OE1, OE2) or aspartate (OD1, OD2).

### Computational calculation of the Gibbs free energy of variant

The targeted residues of the introduced salt bridges in the structure of LiPH8 were applied to the calculation of the energy required for mutation supplemented by the Discovery Studio Client package 4.1. The pH-dependent mode was used in the calculation, in which integration obtained the electrostatic energy over the proton-binding isotherms, as derived from the partial protonation of the sites of titration [29]. The selected mutations were defined as having a stabilizing effect when the changes in Gibbs free energy upon mutations were less than − 0.5 kcal/mol at certain pH values. In contrast, destabilizing effects were assigned for unselected protein variants when the Gibbs free energy due to mutation was higher than 0.5 kcal/mol at specific pH values.

### Acidic pH stability investigation

The enzymes were incubated at pH 2.5 in 0.1 M Britton–Robinson (BR) buffer at 25 °C. The residual activities were assessed by measuring the oxidation of 189 µM ABTS in the presence of 250 μM of H_2_O_2_ in BR buffer (0.1 M, pH 3.0). Activity was recorded at 420 nm within 1 min with a coefficient value *Ɛ*_420nm_ = 36.7 mM^−1^ cm^−1^. The data were fitted to first-order plots and analyzed for the first-order rate constants (*k*_*d*_), which were determined by the linear relationship of the natural logarithm (ln) of the residual activity versus the incubation time (min). The following equation was used to calculate the time required for the residual activity to be reduced to half (*t*_1/2_) of the enzyme’s initial activity at the selected pH value:

$$t_{1/2} = \frac{\ln 2}{{k_{d} }}$$

### Kinetic and substrate consumption studies

To obtain the steady-state kinetic parameters, oxidation was performed with veratryl alcohol (VA). Kinetic investigations of VA were conducted at concentrations ranging from 50 to 2000 µM VA in the presence of 0.02 µM enzyme. The reaction was initiated by adding H_2_O_2_ at a fixed concentration of 250 µM at 25 °C. Absorbance at 310 nm was recorded by a spectrophotometer within the first 30 s of the oxidation reaction and was correlated with the amount of veratraldehyde (VAD) that formed as a degradation product using an extinction coefficient of 9.3 mM^−1^ cm^−1^.

The net oxidation rate was evaluated by examining the amount of consumed substrate in the presence of enzyme and H_2_O_2_ after subtracting the value measured in the presence of H_2_O_2_ alone. The reported data are the mean of triplicate experiments. Steady-state kinetic parameters were obtained from a rearrangement of the Hanes–Woolf plot from the Michaelis–Menten equation.

### Long-term reaction with VA and the model dimeric lignin

The consumption of VA and dimeric lignin catalyzed at pH 2.5 by LiPH8 over time was determined using high-performance liquid chromatography (HPLC). In the presence of 4000 μM substrate, 1 μM and 5 μM enzymes were reacted with VA and dimeric lignin, respectively. The reaction was initiated by feeding H_2_O_2_ at the rate of 150 μM/15 min at 25 °C. At specific time points, an aliquot of the reaction solution was removed and immediately quenched by adding concentrated NaOH. The remaining amount of substrate was detected by high-performance liquid chromatography (HPLC) under previously reported conditions [30].

### pH-Dependent thermal melting profiles

The melting temperature values (*T*_*m*_) of native and variant LiPH8 were determined over a pH range of 2.0–5.0 (BR buffer system, 50 mM) using the differential scanning fluorimetry method. The basic scheme of a thermal shift assay involves incubation of natively folded proteins with SYPRO Orange dye, followed by analysis with a QuantStudio™ 3 Real-time PCR system (The Applied Biosystems Corp. USA).

## Results

### Rational design of LiPH8 variants for improving acid stability by introducing new ionic salt bridges

**Fig. 1:**
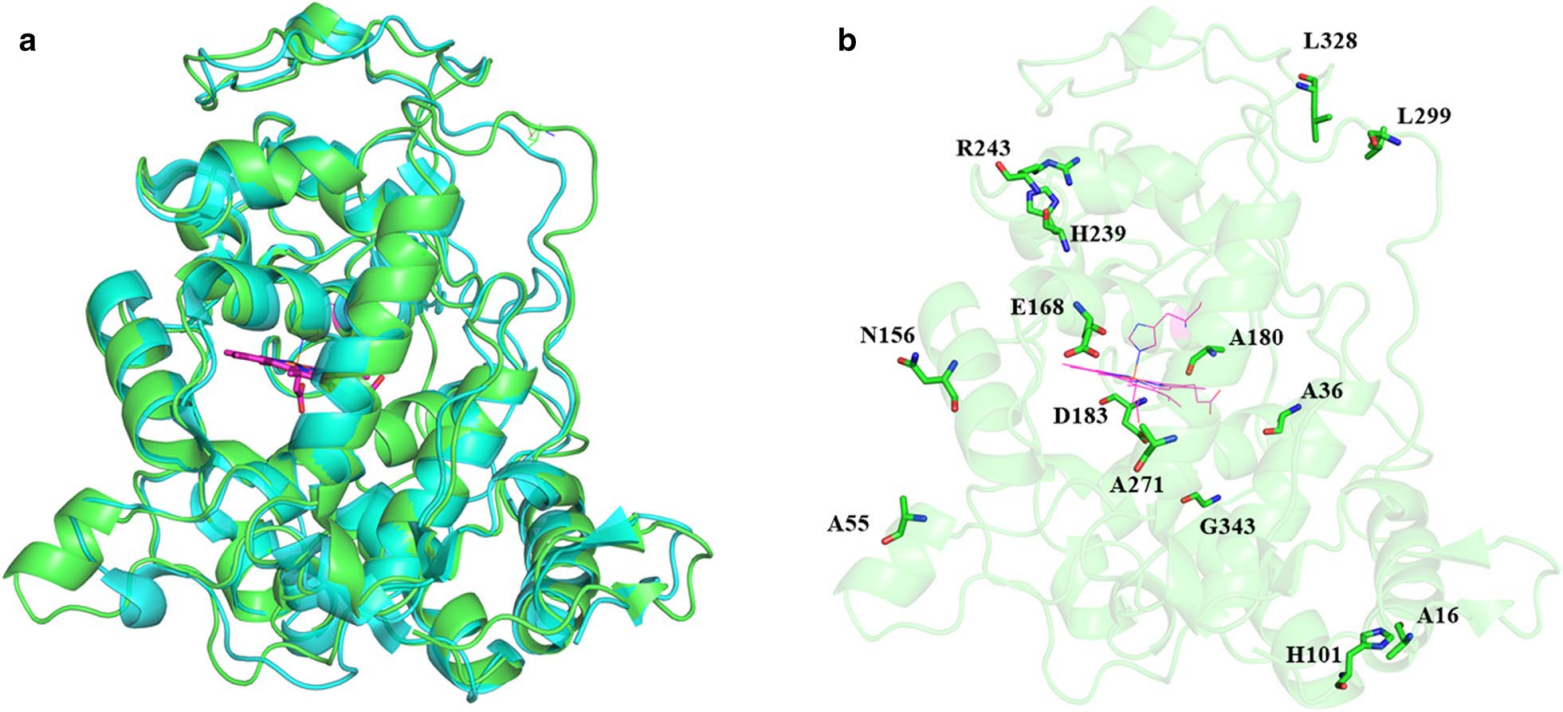
Structural alignment of MnP6 from *C. subvermispora* (PDB 4ZCN, cyan) and LiPH8 from *P. chrysosporium* (PDB 1B80, green) (**a**) and the homologous positions with amino acids that were non-favorable for salt bridge formation in the LiPH8 structure (**b**)

As both MnP6 from *C. subvermispora* and LiPH8 from *P. chrysosporium* are members of the peroxidase family, MnP6 and LiPH8 had 42.79% and 56.22% of amino acid sequence identity and similarity, respectively. Their protein structures also shared a common structural scaffold, with a RMSD of 0.712 Å (Fig. 1a). The high degree of homology in both protein sequence and structure between the two enzymes strongly suggests that they share homologous salt bridge motifs to retain their stable dynamic conformation. MnP6 exhibits high stability under acidic conditions, such as pH 2.0 [4], which may be due to the occurrence of salt bridges and a hydrogen bond network on the protein surface [29]. We executed the MD simulation of the solvated MnP6 structure and searched for existing salt bridges on the structure of MnP6 to determine the contribution of salt bridges to the enhanced pH stability. A potential salt bridge is an interaction that is defined as the interaction between positively charged residues, such as Lys, Arg, and His, and negatively charged residues, such as Asp and Glu, where the distance between them is within 4 Å [11] during 1 ns of production MD simulation. Analysis of potential energy and RMSD is shown in the Additional file 1: Figure S1.

A total of 14 salt bridges were observed in the structure of MnP6 at the desired pH of pH 2.5 (Additional file 1: Table S1). Superimposing the crystal structures of MnP6 and LiPH8 indicated that six salt bridges are conserved in LiPH8. Eight pairs of amino acid residues in the primary structure of LiPH8 were incompatible with the salt bridge formation (Fig. 1b). To improve the stability of LiPH8 under acidic conditions, mutations for salt bridge formation were targeted at these homologous positions.

Furthermore, we calculated the pH-dependent Gibbs free energy of these targeted variants to minimize the unexpected impact of the mutations on the overall stability of the protein structure. Only three predicted mutated sites, A16E, A55R/N156E, and H239E, were estimated to provide a stabilizing effect on the overall protein structure compared to native LiPH8 [based on their calculated Gibbs free energies depending on variable pH conditions (Table 1, Additional file 1: Figure S2)]. These three variants, as well as variants that combined these mutations, were prepared. Their stability under targeted acidic pH conditions was determined and compared with that of native LiPH8.

**Table 1:** Rationale design of salt bridges in LiPH8 at low pH

Potential candidates for introduced salt bridges in LiPH8	Effect of mutation based on calculation of mutation energy
A16**X**-H101	Stabilizing effect: A16E
A36**X**-H39/A180**X**	Destabilizing effect
A55**X**-N156**X**	Stabilizing effect: A55R/N156E
D183-G343**X**	Destabilizing effect
E168-A271**X**	Destabilizing effect
Q189**X**-R337	Destabilizing effect
H239**X**-R234	Stabilizing effect: H239E
L328**X**-L299**X**	Destabilizing effect

### Stability of LiPH8 variants under acid pH conditions

Purified LiPH8 variants exhibited a similar UV–visible absorption spectrum to that of native LiPH8, showing a relative maximum at 409 nm (Soret band) (Additional file 1: Figure S3), which demonstrated that the heme was appropriately incorporated into all the recombinant LiPH8 proteins.

The stabilities of native and variants were evaluated by incubation at pH 2.5. The residual activity was determined using ABTS as the substrate. The half-life of each variant was determined and compared to that of native LiPH8. The results revealed that all three single variants, A16E, A55R/N156E, and H239E, in which the calculated Gibbs free energy changes upon their mutation were estimated to give stabilizing effects, were significantly more stable than native LiPH8 under acidic pH conditions. A 12.5-fold improvement in stability at pH 2.5 was observed for the H239E variant compared to native LiPH8 (Table 2). The other variants, such as Q189D, A36E/A180K, and L238D/L299K, which were in silico predicted as destabilizing effects or neutral effects, led to lower stabilities compared with native LiPH8 (Table 2).

**Table 2:** Stability of LiPH8 variants under acidic pH conditions

LiPH8 variant	Half-life time *t*_1/2_ (min)	Change of Gibbs free energy ΔΔ*G*_mut_ (kcal/mol)
Native LiPH8	9.9 ± 0.8	
LiPH8 variants with in silico prediction as stabilizing effect^a^		
A16E	70.6 ± 4.7	− 0.99
A55R/N156E	21.5 ± 3.5	− 0.58
H239E	117.7 ± 24.5	− 3.58
A16E-A55R/N156E	19.7 ± 0.4	− 0.68
A16E-H239E	47.8 ± 3.7	− 2.41
A55R/N156E-H239E	113.54 ± 17.2	− 1.46
A16E-A55R/N156E-H239E	53.0 ± 5.8	− 2.17
LiPH8 variants with in silico prediction as neutral/destabilizing effect^a^		
A36E/A180K	6.8 ± 1.0	0.22
Q189D	4.5 ± 0.6	0.95
L328D/L299K	13.0 ± 3.7	2.38

^a^ Effect of mutation is defined as a stabilizing effect, neutral effect and destabilizing effect when the Gibbs free energy change upon mutation, ΔΔ*G*_mut_ (kcal/mol), is less than − 0.5, from − 0.5 to 0.5 and higher than 0.5, respectively

We introduced combinations of multiple salt bridges in LiPH8 variants, and the half-life times of these variants were measured at pH 2.5. However, the combination did not exhibit an increased improvement of the half-lives compared to the introduction of a single salt bridge (Table 2).

### Catalytic properties of acid-stable LiPH8 variants

There can be a trade-off between enzyme stability and catalytic activity, so we characterized the catalytic properties of the LiPH8 variants using a typical high-redox-potential substrate of lignin peroxidase (VA) and lignin dimeric model (VE dimer) to investigate their potential application for lignin refinery. The steady-state kinetics of VA oxidation were studied at pH 2.5 and compared with that of native LiPH8 (Table 3). Oxidation of high-redox-potential substrates, such as VA, is mainly catalyzed by the surface-active site Trp171 and its surrounding residues [31]. The trade-off between enzyme stability and activity has been frequently observed in protein engineering studies [32]. However, in this study, we showed that the introduction of noncovalent interactions, such as salt bridges, did not significantly perturb enzyme activity. We found that the A55R/N156E LiPH8 variant retained relatively efficient catalytic activity towards VA. In contrast, the LiPH8 variants A16E and H239E exhibited slightly lower activity compared to native LiPH8. Interestingly, when multiple salt bridges were introduced into LiPH8, all the mutated variants exhibited increased catalytic efficiency for oxidizing VA at pH 2.5. In particular, the activity of variant A55R/N156E-H239E was 1.9-fold more significant than native LiPH8.

**Table 3:** Kinetic parameters for oxidation of veratryl alcohol by native enzyme and variants at pH 2.5

Variant	*K*_*M*_ (μM)	*k*_cat_ (s^−1^)	*k*_cat_/*K*_*M*_ (s^−1^ mM^−1^)
Native LiPH8	121.0 ± 7.9	10.0 ± 0.4	82.8 ± 10.3
LiPH8 variants with the introduction of a single salt bridge			
A16E	57.2 ± 5.9	3.4 ± 0.5	58.6 ± 2.7
A55R/N156E	141.1 ± 33.6	12.6 ± 0.8	92.4 ± 27.8
H239E	90.6 ± 11.4	4.9 ± 0.4	54.5 ± 10.8
LiPH8 variants with the introduction of combined salt bridge			
A16E-A55R/N156E	45.4 ± 9.5	5.5 ± 0.1	123.5 ± 25.0
A16E-H239E	53.4 ± 3.4	7.8 ± 0.0	145.5 ± 8.9
A55R/N156E-H239E	59.8 ± 0.9	9.4 ± 0.1	157.5 ± 2.6
A16E-A55R/N156E-H239E	56.8 ± 1.6	7.6 ± 0.1	133.3 ± 3.7

**Fig. 2:**
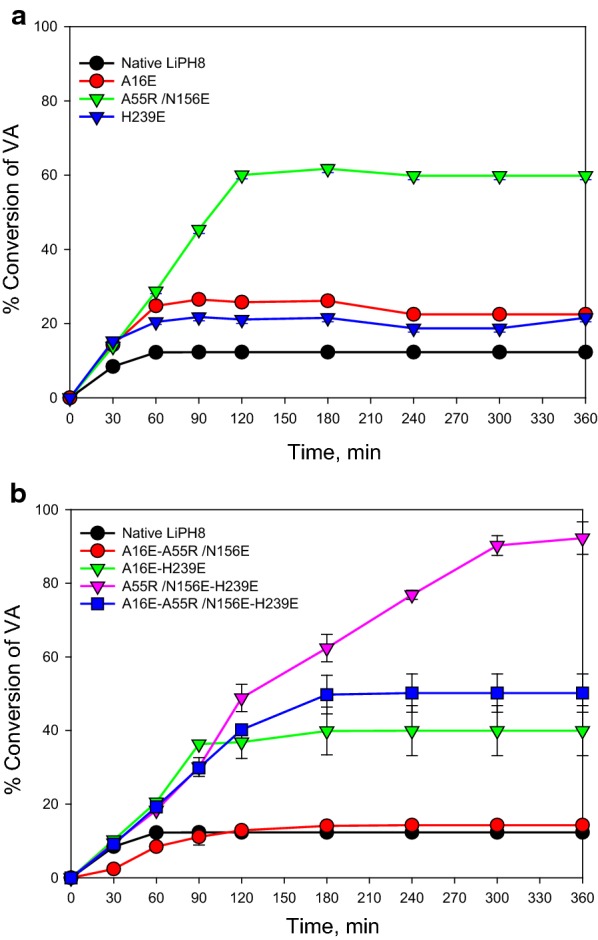
Conversion of VA by native LiPH8 and its variants with introduction of a single salt bridge (**a**) and combined salt bridges (**b**). The oxidation reaction was carried out in 0.1 M BR buffer, pH 2.5 with 4 mM VA and 1 μM native LiPH8 or variants, in which H_2_O_2_ was fed at a rate of 150 μM/15 min at 25°C

In addition to the steady-state kinetic characterization, the long-term catalytic reaction with VA as substrate at an acidic pH was also monitored for native and mutated variants of LiPH8 (Fig. 2). The combination variant A55R/N156E, harboring the new single salt bridge, showed the highest efficiency of VA conversion, which reached approximately 60% after 2 h. In contrast, although the variant H239E exhibited markedly higher stability at an acidic pH compared to native LiPH8, it did not show improved long-term catalysis of VA oxidation. The combination mutations of A55R/N156E with H239E demonstrated a synergistic effect in both acid stability and long-term catalytic activity. The combined variant A55R/N156E-H239E exhibited a 9.9-fold increased efficiency for VA oxidation (approximately 90.2%) compared to native LiPH8 after a 6-h reaction.

**Fig. 3:**
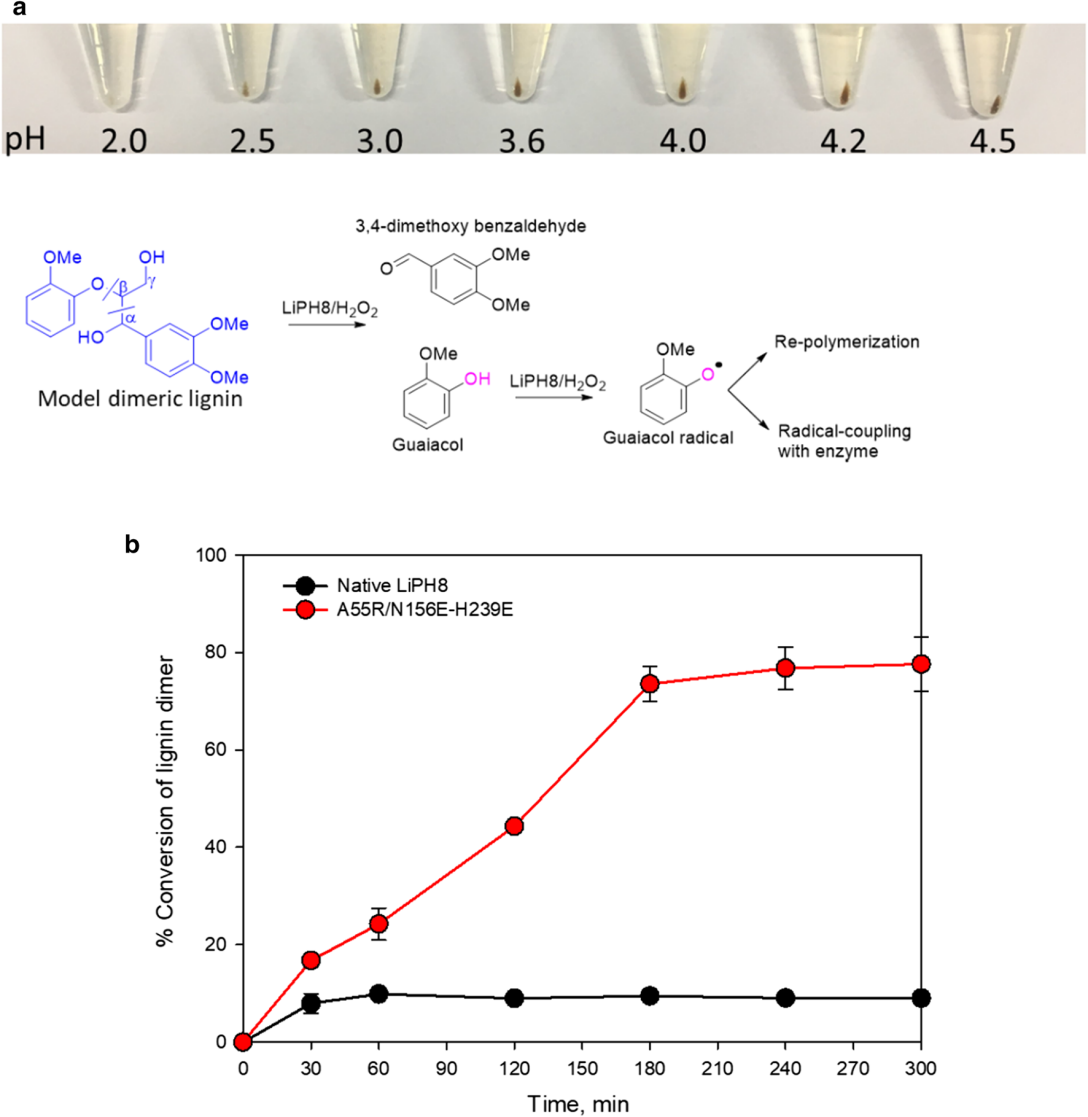
Effect of pH on the repolymerization of the released phenolic product from degradation of dimeric lignin (**a**) and conversion of the model dimeric lignin by native and variant A55R/N156E-H239E at pH 2.5 (**b**). The oxidation reaction was carried out in 0.1 M BR buffer, pH 2.0 to 4.5 with 4 mM lignin dimer and 5 μM native LiPH8 or variants, in which H_2_O_2_ was fed at a rate of 150 μM/15 min at 25 °C

Repolymerization of phenolic products is a barrier in in vitro lignin degradation using oxidative catalysts [33]. In this work, recombination of phenolic products released from VE dimeric lignin occurred simultaneously at a significant rate under pH 3–4.5 compared with reaction at pH ≤ 2.5 (Fig. 3a). The conversion of VE dimer by engineered LiPH8 at pH 2.5 approached approximately 76.6%, which showed 7.8-fold enhancement compared to native LiPH8, with a decreased repolymerization (Fig. 3b).

### Structural elucidation of extremely stable LiPH8 variant

The crystal structure of the variant A55R/N156E-H239E LiPH8 was solved; this variant showed both enhanced acidic pH stability and long-term catalytic activity. The statistics of the crystal structure are summarized in Table 4. Subsequently, structural analyses of native and the variant proteins were performed to investigate how the introduced mutations affected the thermostability of the enzyme. Structural changes were restricted to the regions where the target salt bridges were constructed.

**Table 4:** Data collection and structural refinement statistics

	LiPH8 variant A55R/N156E-H239E
PDB code	6A6Q
Data collection	
Wavelength (Å)	0.97934
Unit cell (*a*, *b*, *c; α, β, g*) (Å; °)	41.2, 99.6, 48.3; 90.0, 113.9, 90.0
Space group	*P2*_*1*_
Solvent content (%)	66.29
Protein chains in AU	1
Resolution range (Å)	50.00–1.66
Highest resolution shell (Å)	1.69–1.66
Unique reflections	40,502
Redundancy	3.2 (2.6)
Completeness (%)	97.7 (94.4)
*R*_merge_ (%)	7.8 (29.6)
Average *I*/*σ*(*I*)	32.1 (5.1)
CC (1/2)	98.5 (80.3)
Refinement	
Resolution range (Å)	29.72–1.67
*R*_work_ (%)	14.2
*R*_free_ (%)	17.1
Mean *B* value (Å^2^)^a^	21.2
RMS deviation bond lengths (Å)	0.016
RMS deviation bond angles (°)	2.0662
Number of amino acid residues	351
Number of water molecules	249
Ramachandran plot	
Most favored regions (%)	97.9
Additional allowed regions (%)	1.8
Outliers (%)	0.3

^a^ The mean *B* value is for both protein atoms and the solvent molecules

**Fig. 4:**
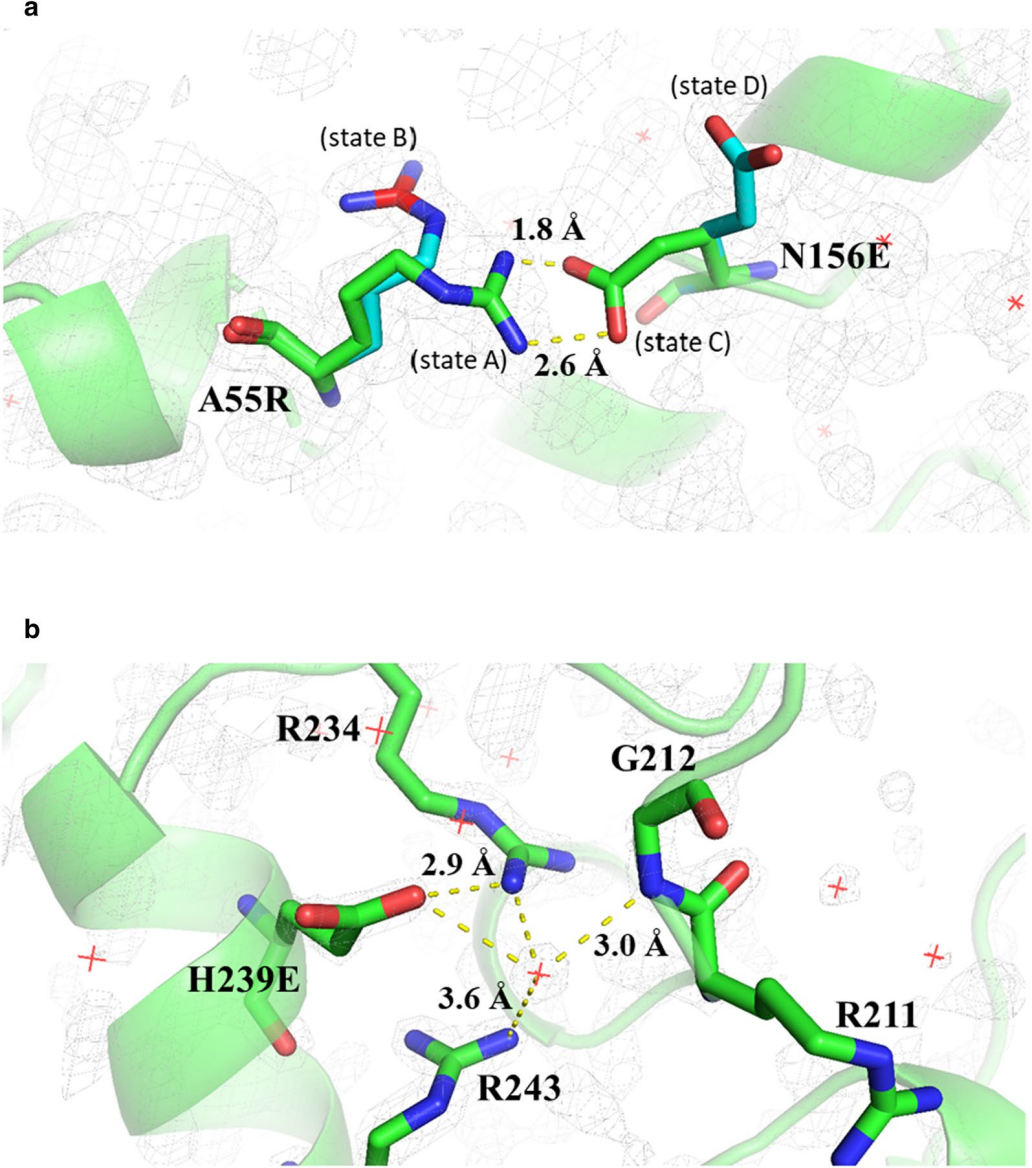
Side-chain conformations of the salt bridges introduced in the variant A55R/N156E-H239E, resolution 1.67 Å. **a** Residue Arg55 has two alternate locations, state A and B, with 0.5 occupancy for each state; and residue Glu156 includes two states, C and D, sharing equal occupancies (0.5). **b** Rigid hydrogen bonding and salt bridges between residues surrounding amino acid Glu239

The crystal structure of the variant A55R/N156R-H239E LiPH8 showed the formation of salt bridges, as expected. The side-chains of A55R and N156E had two alternate locations on the electron density map (Fig. 4a). By contrast, rigid hydrogen bonding and a network of salt bridges were found between residues surrounding the introduced H239E mutation (Fig. 4b). These observations are consistent with the experimental data, which showed that the H239E mutation contributed more to the enhanced acidic pH stability in LiPH8 (*t*_1/2_ ~ 117.7 min) than the salt bridges formed by the A55R/N156E mutations (*t*_1/2_ ~ 21.5 min) (Table 2).

**Fig. 5:**
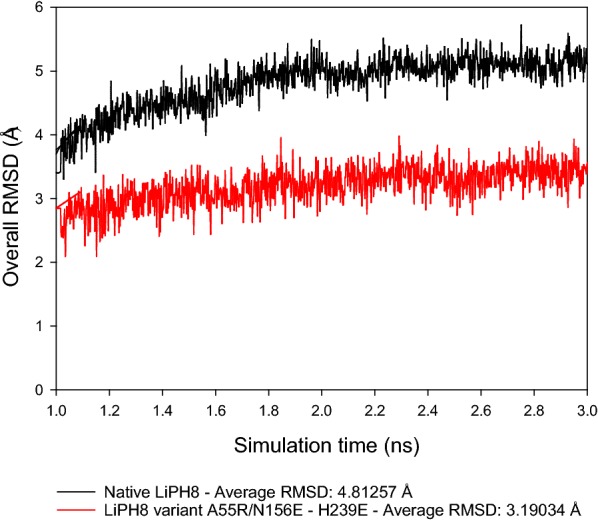
RMSD changes of an LiPH8 variant for the last 2 ns of the MD simulation at 300 K

Furthermore, MD simulation at 300 K was performed to investigate the flexibility differences between the structures of native LiPH8 and its variant. The average RMSD at 300 K for the overall structure of native LiPH8 (RMSD: 4.81257 Å) was also higher than that measured for the A55R/N156E-H239E (RMSD: 3.19034 Å) (Fig. 5). In other words, introducing salt bridges reinforced the enhanced rigidity of the variant A55R/N156E-H239E LiPH8 compared to native LiPH8.

**Fig. 6:**
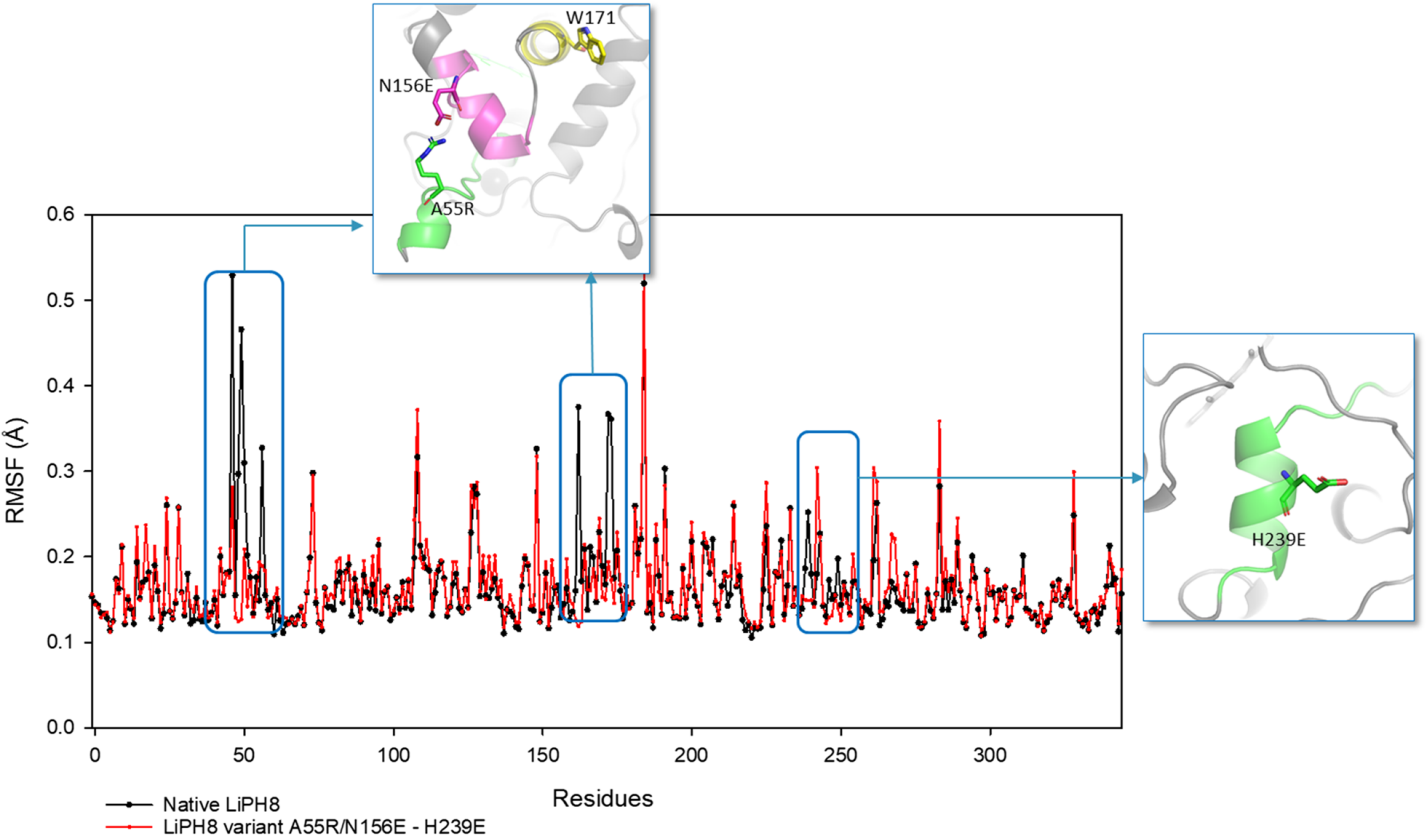
The per-residue flexibility of native (black line, dotted-scatter) and variant A55R/N156E-H239E (red line, dotted-scatter)

Per-residue RMSF was also analyzed at room temperature in native LiPH8 to further evaluate the impact of mutations on the structural flexibility of the enzyme, which was higher than that of the A55R/N156E-H239E LiPH8 variant. Increased flexibility was observed not only at the introduced salt bridges, but also at the alpha helices close to the mutated sites (Fig. 6). This result indicated that the interactions between charged residues kept their adjacent and distant helices more stable, while retaining activity under lower pH. We also found that a helix containing the active site Trp171 showed a significant decrease in fluctuation (yellow-colored helix, Fig. 6). The thermodynamic stability of this active helix was strengthened at low pH. As a result, this variant more efficiently catalyzed the oxidation of VA and dimeric lignin than native LiPH8.

## Discussion

In this study, the combination of searching for stable salt bridges under desired conditions and calculating the stability of the structures generated by mutation was a good approach for designing promising candidates to improve the acidic pH stability of LiP. Instead of a fixed atomic charge, the pH-dependent electrostatic energy term of native and mutated structures in both the folded and unfolded states was studied here [29]. In other words, the charge states of titratable acid and basic residues at targeted positions were weighted in the sum of Gibbs free energy terms. As a result, all three candidates for mutation (i.e., whose Gibbs free energy changes were lower than − 0.5 kcal/mol) exhibited higher acid stability compared with native (Table 2). To test our rational approach for designing salt bridges, variants including: Q189D, A36E/A180K, and L238D/L299K, were in silico designed to demonstrate destabilizing or neutral effects based on Gibbs free energy changes, were also prepared for stability testing. Under the same conditions, the experimental data showed that these mutations exhibited neutral effects, and some variants even led to lower stabilities compared to native LiPH8 (Table 2). These results indicate that the approach of using pH-dependent calculations of the Gibbs free energy change upon mutation to evaluate targeted variants is useful for the generation of acid-stable and active variants.

Furthermore, adding salt bridges not only enhanced acid stability, but also accelerated the thermal stability of the enzyme. *T*_*m*_ was assessed at different pH values and the variant proteins had increased *T*_*m*_: native LiPH8 had a *T*_*m*_ of 49 °C, whereas the A55R/N156E-H239E variant LiPH8 had a *T*_*m*_ of 51.0 °C at pH 5. However, at pH 2.5, some of the salt bridges dissociated, which narrowed the gap in *T*_*m*_ values compared to the gap at pH 5 (Additional file 1: Figure S4). Salt bridges may not be strong enough to retain the protein structure at elevated temperatures. Introduction of disulfide bonds as a robust interaction at this region may help to maintain protein structure, not only under acidic conditions, but also at high temperature.

Compared to LiPs and VPs, many characterized MnPs from other white-rot fungi, such as MnP5, MnP6, MnP10 and MnP12 from *C. subvermispora* [20] and MnP4 from *P. ostreatus* [4], show remarkable stability under extremely acidic conditions (pH 2.0). In the evolutionary cladogram, the evolution of modern LiPs from MnPs took place by replacing Mn^2+^-binding sites with exposed active tryptophanyl radical sites [34]. VPs may be an intermediate in this evolutionary process, as they utilize both Mn^2+^ ions and VA as mediators [35]. The use of this intermediate possibly leads to an evolutionarily enhanced interaction between LiPs and lignin using a diffusion mechanism of a redox mediator, cationic radical VA. However, this observation also leads to a stability–activity trade-off with lignin under extremely acidic conditions and results in unexpected repolymerization of released phenolic products following degradation of lignin. Herein, with the introduction of salt bridges at the appropriate positions, we recreated an active lignin peroxidase variant (LiPH8 variant A55R/N156E-H239E) from ancient ligninase (native LiPH8) that exhibited remarkable stability under extremely acidic conditions, such as MnPs, and still retained an exposed active site for lignin (Additional file 1: Figure S5). This resurrection was also reported for the engineered VP isozyme 2 from *P. eryngii*, which exhibited improved acid stability by incorporating the conserved basic residues in MnP4 from *P. ostreatus* [36].

Compared to the VPi variant, an introduced salt bridge between residues Ala55Arg and Asn156Glu in LiPH8 was not constructed in an engineered VPi variant (Additional file 1: Figure S6). By contrast, a VPi variant with mutation of His232 to Glu was found at a homologous position to the salt bridges between amino acids Arg234, His239Glu, and Arg243 that was rationally designed for LiPH8 in this study. In this regard, the homologous position was found at Arg242–Asp246–His251 of MnP6 (Additional file 1: Figure S7). The introduced salt bridges between Glu-Arg ionic pairs suggested, according to the calculated Gibbs free energy, enhanced stability of the variants under acidic pH conditions. This result agrees with a previous paper in which the thermodynamic stability between peptides containing different types of salt bridges followed the trend Glu-Arg > Asp-Lys > Glu-Lys at both neutral and acidic pH [37].

## Conclusion

The results obtained in this study demonstrate a compelling and rational approach for resurrecting ancient LiP to acquire stability, as well as activity under extremely acidic conditions. This effective approach might suggest the future of evolutionarily converged LiPs for more effective depolymerization of lignin which in turn enhances their potential application as valuable assets for the lignin biorefinery.

## Additional file

**Additional file 1.** Additional figures and tables.

## Abbreviations

HPLC: high-performance liquid chromatography
BR: Britton–Robinson
MnP: manganese peroxidase
LiP: lignin peroxidase
VP: versatile peroxidase
LiPH8: lignin peroxidase isozyme H8
MnP6: manganese peroxidase isozyme 6
VA: veratryl alcohol
VE dimer: veratrylglycerol β-guaiacyl ether
ABTS: 2,2′-azino-bis-(3-ethylbenzothiazoline-6-sulfonic acid) diammonium salt
MD: molecular dynamics
RMSD: root-mean-square deviation
RMSF: root-mean-square fluctuation
