# Supplementary material for: In silico-designed lignin peroxidase from Phanerochaete chrysosporium shows enhanced acid stability for depolymerization of lignin
Source: Biotechnol Biofuels. 2018 Dec 10;11:325. doi: 10.1186/s13068-018-1324-4 (PMC6287364; doi:10.1186/s13068-018-1324-4)
Supplement: Supplementary file 1 — Additional file 1. Additional figures and tables. [file 13068_2018_1324_MOESM1_ESM.docx]

Addtional file 1

***In silico*–designed lignin peroxidase from *Phanerochaete chrysosporium* shows enhanced acid stability for depolymerization of lignin**

Le Thanh Mai Pham^1^, Hogyun Seo^2^, Kyung-Jin Kim^2*^, Yong Hwan Kim^1*^

From the ^1^School of Energy and Chemical Engineering, UNIST, 50 UNIST-gil, Ulju-gun, Ulsan, 44919, Republic of Korea; ^2^ School of Life Sciences (KNU Creative BioResearch Group), KNU Institute for Microorganisms, Kyungpook National University, Daehak-ro 80, Buk-gu, Daegu, 41566, Republic of Korea

^*^Corresponding author

Prof. Yong Hwan Kim, School of Energy and Chemical Engineering, UNIST, 50 UNIST-gil, Ulju-gun, Ulsan, 44919, Republic of Korea. Email: [metalkim@unist.ac.kr](mailto:metalkim@unist.ac.kr)

Prof. Kyung-Jin Kim, School of Life Sciences (KNU Creative BioResearch Group), KNU Institute for Microorganisms, Kyungpook National University, Daehak-ro 80, Buk-gu, Daegu, 41566, Republic of Korea. Email: kkim@knu.ac.kr

**Table S1: Primers used for mutagenesis**

| **Primer** | **Sequence^a^** | **T_m_ (°C)^b^** |
| --- | --- | --- |
| A16E-F | 5’-AGCTGTTGT*GAA*TGGTTCGATGTTCTG GATGAT-3’ | 64.0 |
| A16E-R | 5’-ATCGAACCA*TTC*ACAACAGCTCGCATCAC-3’ | 64.0 |
| A36E-F | 5’-CAATGCGGT*GAA*GAAGCTCATGAAAGTATCCGT-3’ | 64.0 |
| A36E-R | 5’-ATGAGCTTC*TTC*ACCGCATTGACCGC-3’ | 64.0 |
| A55R-F | 5’-ATCTCCCCG*CGT*ATGGAAGCACAGGGTAA-3’ | 66.3 |
| A55R-R | 5’-TGCTTCCAT*ACG*CGGGGAGATTGCAATACTATC-3’ | 65.0 |
| N156E-F | 5’-CAGATTATC*GAA*CGCGTTAATGATGCAGGCGAA-3’ | 65.0 |
| N156E-R | 5’-ATTAACGCG*TTC*GATAATCTGGTCCACGGTATGG-3’ | 65.0 |
| A180K-F | 5’-TCTGTGGCA*AAA*GTTAACGATGTCGACCC-3’ | 63.0 |
| A180K-R | 5’-ATCGTTAAC*TTT*TGCCACAGAGTGTGCGC-3’ | 64.0 |
| Q189D-F | 5’-CCGACCGTC*GAT*GGTCTGCCGTTCGATAGTA-3’ | 67.0 |
| Q189D-R | 5’-GCAGACC*ATC*GACGGTCGGGTCGACATC-3’ | 68.0 |
| L299K-F | 5’-CCGGGTAAT*AAA*CCGTTCAGCTTTTTCCCG-3’ | 68.0 |
| L299K-F | 5’-GCTGAACGG*TTT*ATTACCCGGGATCGGTTTAGA-3’ | 69.0 |
| L328D-F | 5’-CTGACGACG*GAT*CCGGGTCCGGAAACCT-3’ | 69.0 |
| L328D-R | 5’-GACCCGG*ATC*CGTCGTCAGCGTCGGAA-3’ | 69.0 |

^a^: The mutated sequence is in italics and underlined

^b^: T_m_ was calculated from the primer sequence matched to the template.

**Table S2: Probed salt bridges at pH 2.5 found in MnP6 structure and homologous positions in LiPH8 structure**

| Probed salt bridges  on MnP6 structure | Homologous positions  on LiPH8 structure |
| --- | --- |
| D16-H97 | A16 - H101 |
| D23-H97 | D23 - H101 |
| D104-R129 | D107 - R132 |
| D157-R154 | D160 - R157 |
| D179-K180 | E168 - A271 |
| D186-H344 | Q189 - R337 |
| D246-H251 | H239 – R234 |
| D252-R254 | D244 - R246 |
| D334-K306 | L328 - L299 |
| D363-K180 | G343-D183 |
| E35-R177 | A36 -A180 |
| E153-K54 | A55 - N156 |
| E165-K279 | E168 - A271 |
| E320-H251 | E314 - R243 |

**Figure S1:** MD simulation in the production stage (1 ns) with analysis of potential energy (A) and RMSD (B).

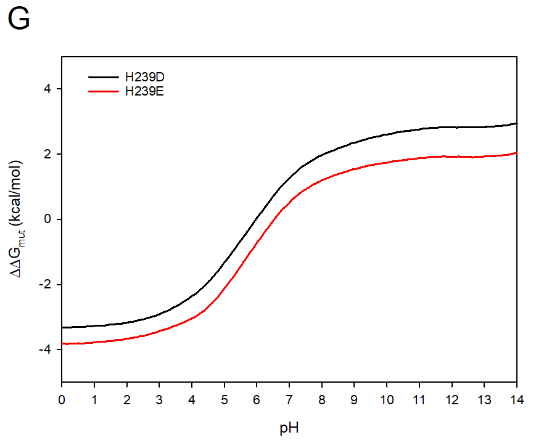

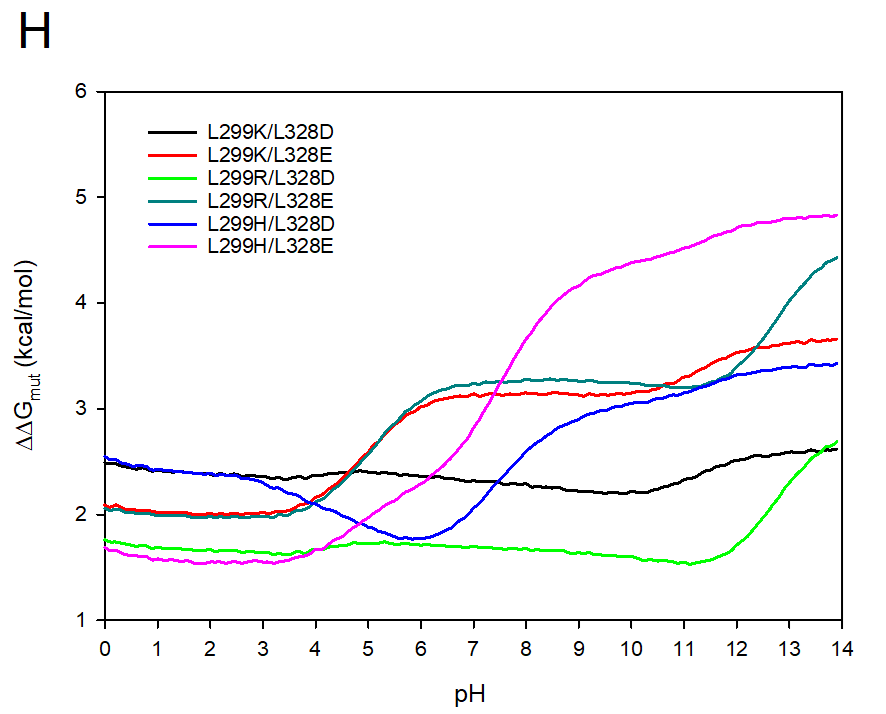


**Figure S2:** Calculation of Gibbs free energy changes upon mutations (kcal/mol) for designed salt bridges in LiPH8 structure: A16**X** - H101 (A), A36**X** - H39/A180**X** (B), A55**X** - N156**X** (C), D183 - G343**X** (D), E168 - A271**X** (E), Q189**X** - R337 (F), H239**X** – R234 (G), L299**X-**L328**X** (H).

**Figure S3:** UV-Vis spectrum of native and variants recorded by spectrophotometer at room temperature. Protein samples were prepared in acetate buffer 10 mM, pH 6.0.

**Figure S4:** pH-dependent determination of T_m_ for native LiPH8 and its variant A55R/N156E-H239E.


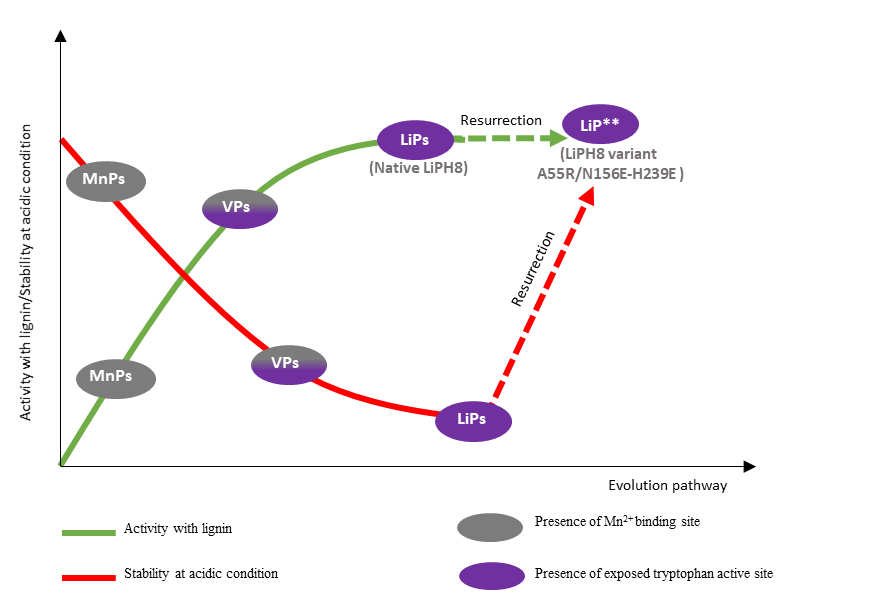


**Figure S5.** Activity-stability trade-off in the evolutionary pathway of ligninases from white-rot fungi and the restoration of a LiP** as LiPH8 variant A55R/N156E-H239E with two introduced salt bridges compared to LiPs (native LiPH8).


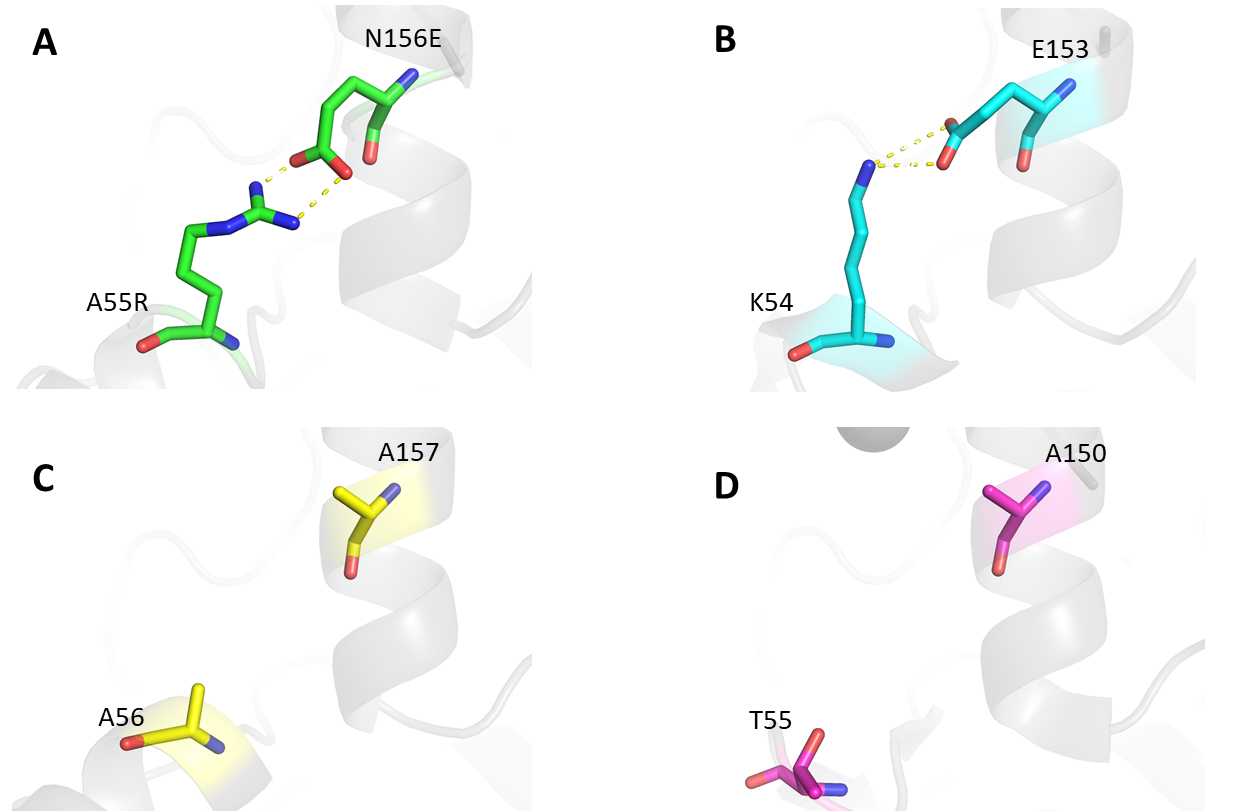


**Figure S6:** Homologous structure analysis of designed salt bridge between residues Ala55Arg - Asn156Glu of LiPH8 variant from *P. chrysosporium* (A, PDB ID: 6A6Q), MnP6 from *C. subvermispora* (B, PDB ID: 4CZN), MnP4 from *P. ostreatus* (C, PDB ID: 4BM1) and VPi variant from *P. eryngii* (D, PDB ID: 5ABN).


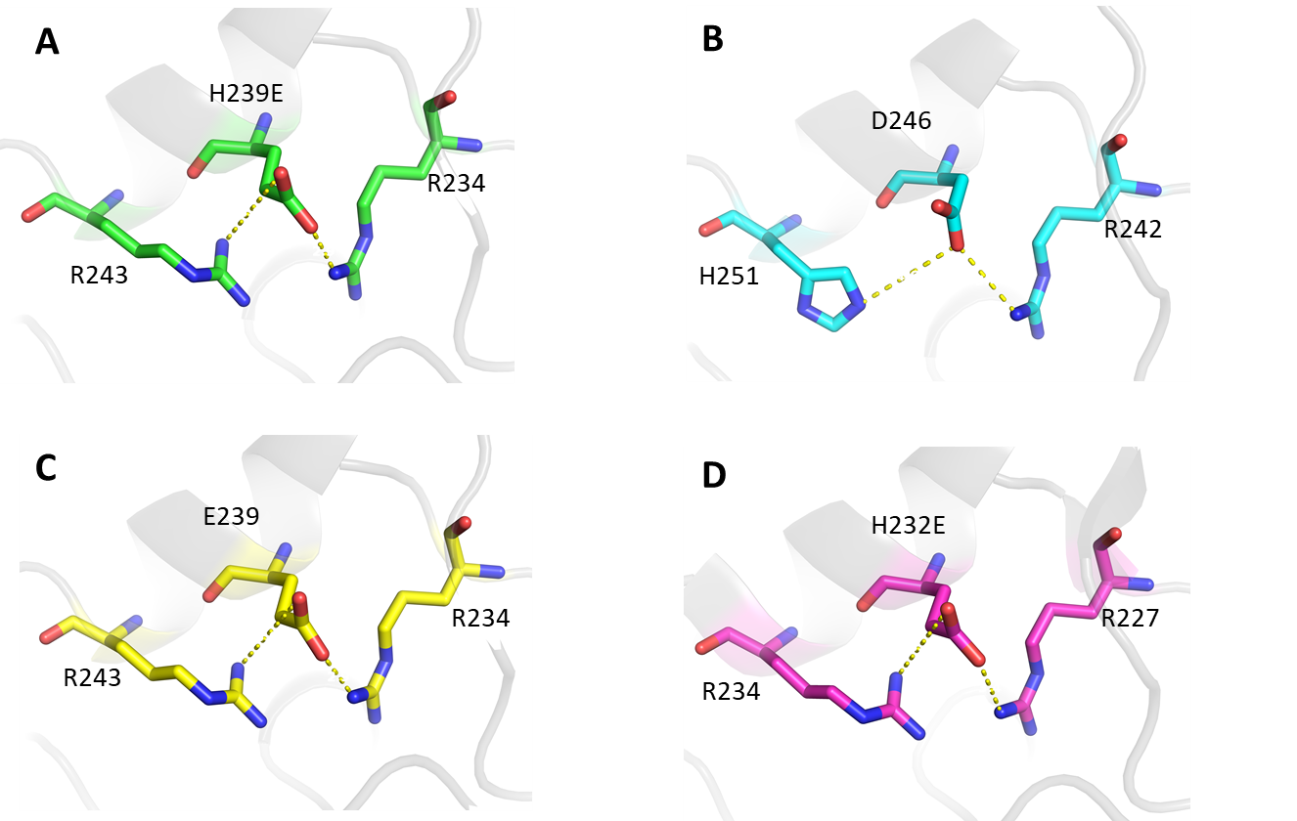


**Figure S7:** Homologous structure analysis of designed salt bridges between Arg234, His239Glu and Arg243 of LiPH8 variant from *P. chrysosporium* (A, PDB ID: 6A6Q), MnP6 from *C. subvermispora* (B, PDB ID: 4CZN), MnP4 from *P. ostreatus* (C, PDB ID: 4BM1) and VPi variant from *P. eryngii* (D, PDB ID: 5ABN).
